# Relationship between SP142 PD-L1 Expression and ^18^F-FDG Uptake in Non-Small-Cell Lung Cancer

**DOI:** 10.1155/2020/2010924

**Published:** 2020-07-20

**Authors:** Long Zhao, Jinjun Liu, Jingyun Shi, Huoqiang Wang

**Affiliations:** ^1^Department of Nuclear Medicine, Shanghai Pulmonary Hospital, Tongji University School of Medicine, 507 Zhengmin Road, Shanghai 200433, China; ^2^Department of Radiology, Shanghai Pulmonary Hospital, Tongji University School of Medicine, 507 Zhengmin Road, Shanghai 200433, China

## Abstract

**Objectives:**

Immune checkpoint blockers constitute the first-line treatment for advanced non-small-cell lung cancer (NSCLC) with ≥50% PD-L1 expression. In NSCLC, PD-L1 positivity is correlated with high ^18^F-fluorodeoxyglucose (^18^F-FDG) uptake. However, these studies only included patients undergoing surgical resection, almost all in their early stages. Moreover, differences in ^18^F-FDG uptake between NSCLC with high (≥50%) and low (49%) PD-L1 expression remain unknown. We aimed to investigate the association between metabolic parameter ^18^F-FDG uptake and PD-L1 expression status in NSCLC patients.

**Methods:**

From February 2017 to June 2018, 428 consecutive NSCLC patients who underwent ^18^F-FDG positron emission tomography/computed tomography (PET/CT) and SP142 PD-L1 expression analysis were retrospectively assessed. The association between clinicopathological characteristics and PD-L1 expression was examined.

**Results:**

The frequency of PD-L1-positive tumors was 38.1% (163/428), 28.5% (91/319), and 64.2% (61/95) for NSCLC, adenocarcinoma (ADC), and squamous cell carcinoma (SCC), respectively. Maximal standard uptake (SUVmax) was significantly higher in PD-L1-positive than in PD-L1-negative NSCLC (*p* < 0.0001), ADC (*p* < 0.0001), and SCC (*p*=0.006). SUVmax was significantly higher in NSCLC (*p*=0.001) and ADC (*p*=0.003) with high rather than low PD-L1 expression. The receiver operating characteristic curve yielded area under the curve values of 0.726 (95% CI, 0.679–0.774, *p* < 0.0001), 0.694 (95% CI, 0.634–0.755, *p* < 0.0001), and 0.625 (95% CI, 0.513–0.738, *p*=0.044) for NSCLC, ADC, and SCC, respectively.

**Conclusion:**

^18^F-FDG tumor uptake is strongly, positively correlated with PD-L1 expression in NSCLC and significantly differs between high and low PD-L1-expressing individuals.

## 1. Introduction

During the past decades, owing to heavy smoking and air pollution, lung cancer has become the most common cancer and the leading cause of cancer death in China [1–4]. The 5-year survival rate of lung cancer is 20.2%, and that of patients with advanced disease is significantly worse [5]. Nonetheless, in the last decade, immunotherapy has emerged oncotherapeutic modality. Positive results have been obtained in the treatment of non-small-cell lung cancer (NSCLC) [6–8], which have drastically revolutionized cancer therapy. The immune checkpoint status (programmed death ligand-1, PD-L1) test is an effective method to identify patients who can benefit from immunotherapy. PD-L1-positive patients with advanced NSCLC, specifically patients with high PD-L1 expression, have better survival [7, 8]. Analysis of the immune checkpoint status has become a standard of care, and tumor tissues aspirated during biopsy or intraoperatively resected are usually obtained from patients with tumors. However, owing to the inherent limitations of invasive procedures in patients with advanced tumors, tissues are not always readily available or may be difficult to access, resulting in sampling errors or insufficient material. Therefore, a complementary noninvasive biomarker for predicting PD-L1 expression in advanced NSCLC would be highly useful.

Recent studies have reported the predictive value of ^18^F-fluorodeoxyglucose positron emission tomography/computed tomography (^18^F-FDG PET/CT) to determine the PD-L1 expression status in cancer patients; however, their results are not consistent [9–22]. Hence, tumor FDG uptake is generally higher in PD-L1-positive malignancies [9–15, 19, 22]. However, Chen et al. reported contrasting findings, reporting an inverse correlation between metabolic tumor volume (MTV) and total lesion glycolysis (TLG) with PD-L1 expression [20]. Three other studies have reported the absence of an association between the maximal standard uptake value (SUVmax) and PD-L1 expression status [16, 17, 21]. For clinical purposes, it is important to know if the SUVmax can predict the PD-L1 expression status and identify patients who could potentially benefit from PD-L1/PD-1-targeted therapies. In addition, patients with NSCLC undergoing surgical resection have been previously assessed in these studies, almost all in their early stages. However, clinical studies have reported that antibodies targeting PD-L1 are an efficacious treatment alternative for patients with advanced NSCLC, who have ≥1% PD-L1 expression [6–8]; studies including more patients with advanced disease would be persuasive and clinically significant in assessing the association between ^18^F-FDG uptake and PD-L1 expression in NSCLC. Furthermore, pembrolizumab has been approved as a single agent for the first-line therapy for patients with advanced NSCLC and high PD-L1 expression (≥50%). To our knowledge, no studies have investigated the differences in ^18^F-FDG uptake between NSCLC patients with high PD-L1 expression (≥50%) and low PD-L1 expression (1%–49%). In this study, we retrospectively assessed patients with NSCLC, including more patients with advanced disease, and investigated the association between ^18^F-FDG PET/CT metrics and PD-L1 expression status.

## 2. Materials and Methods

### 2.1. Study Design and Patient Cohort

From February 2017 to June 2018, we retrospectively reviewed all NSCLC patients who underwent PET/CT by searching our hospital's database at the Department of Nuclear Medicine. This study was approved by the Institutional Review Board of our hospital. Patients with NSCLC were included in accordance with the following criteria: (1) available pathology reports with newly diagnosed NSCLC, (2) patients not receiving previous chemotherapy or radiotherapy before PET/CT and sampling for PD-L1 analysis, (3) no history of malignancy, (4) an interval between sampling and PET/CT shorter than 1 month, and (5) presence of a well-defined lesion. We retrospectively reviewed clinical data of these patients, including sex, age at diagnosis, pathological tumor-lymph node-metastasis stage (in accordance with the International Association for the Study of Lung Cancer, 8th TNM Lung Cancer Staging System), and smoking status. Smoking status was divided into two categories: nonsmoker and smokers (including current and previous smokers).

### 2.2. ^18^F-FDG PET/CT Acquisition and Analysis

At the time of diagnosis, all patients underwent ^18^F-FDG PET/CT scans (Biograph Mct64, Siemens, Erlangen, Germany). Before the examination, the patients were fasted for 4–6 h to maintain serum glucose levels below <11.1 mmol/L. The ^18^F-FDG injection volume was 0.10～0.15 mCi/kg. After injection, all patients rested for 1 h and were additionally administered at least 500 mL of water. Imaging was carried out at six or seven bed positions from the base of the skull to the midthighs [15]. Visual analysis and PET/CT measurements were independently performed by two experienced nuclear medicine physicians (W. HQ and L.Q.) blinded to the PD-L1 expression status and the clinical data, and interpretations were reached through consensus. The SUVmax values of the primary tumors were determined.

### 2.3. PD-L1 Histopathological and Mutational Analyses

Hematoxylin and eosin-stained slides and immunostained slides upon primary diagnosis (performed at the Department of Pathology in our hospital) were available for all patients. For immunostaining for SP142 (Ventana Medical Systems Inc.), the specimens were formalin-fixed, paraffin-embedded, sectioned, and stained in accordance with standard clinical operating procedures. The proportion of PD-L1-positive tumor cells was determined by calculating the percentage of stained tumor cells. Positivity was defined as a proportion of PD-L1-positive tumor cells >1%. Proportions of PD-L1-positive tumor cells ≥50% and 1–49% were defined as indicative of high and low expression, respectively.

We retrospectively reviewed the mutational status of the epidermal growth factor receptor (EGFR, exons 18–22) gene in all patients. Tumor samples were obtained through surgical resection or biopsy. EGFR mutations were analyzed using the amplification-refractory mutation system. Cycle sequencing of the purified PCR products was performed using the ADx Mutation Detection Kits (Amory, Xiamen, China) in accordance with the manufacturer's instructions.

### 2.4. Statistical Analysis

The clinicopathological characteristics and PET/CT findings are presented as continuous variables. Univariate and multivariate logistic regression analyses were performed to assess the association between the PD-L1 expression status and each of the considered factors, i.e., age, sex, smoking status, tumor stage, lymph node metastases, and EGFR status. The association between quantitative continuous variables (including SUVmax and maximum diameter) and PD-L1 expression status was also investigated using the Mann–Whitney *U* test. A *p* value <0.05 was considered statistically significant. The cutoff value of SUVmax was determined through receiver operating characteristic (ROC) curve analysis. All data were statistically analyzed using a software program (SPSS version 21.0; SPSS, Chicago, IL, USA).

## 3. Results

### 3.1. Patient Characteristics

**Figure 1:**
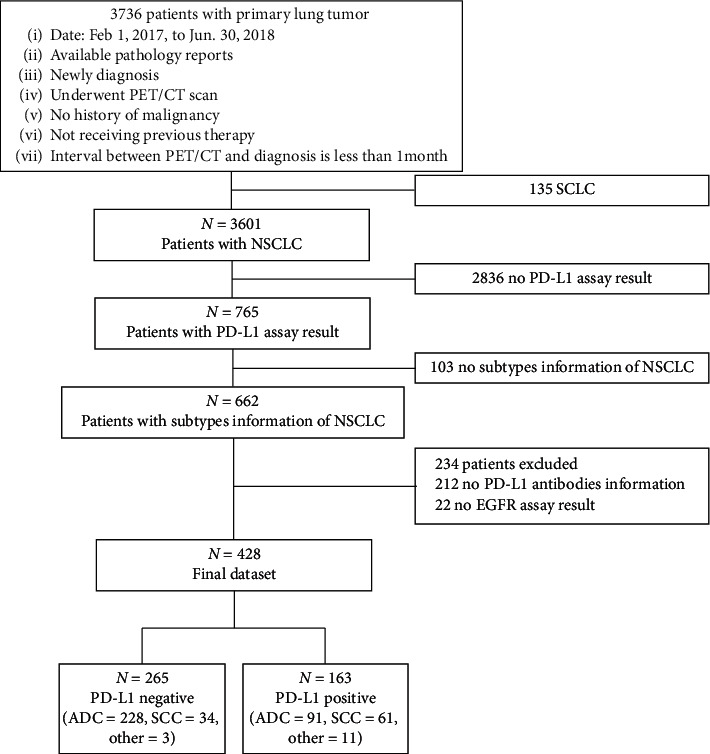
Flowchart illustrating the selection of the study population.

In total, 428 consecutive patients with NSCLC were identified (Figure 1 outlines additional details of the study population cohort selection). Detailed clinicopathological information is presented in Table 1. Three hundred and nineteen patients (74.5%) had pulmonary adenocarcinoma (ADC), 95 (22.2%) pulmonary squamous cell carcinoma (SCC), and 14 (3.3%) other types of lesions (5 sarcomatoid carcinomas, 4 large cell carcinoma, 3 adenosquamous carcinoma, and 2 lymphoepithelioma-like carcinoma). PD-L1 expression in biopsy specimens obtained from 35 patients and resection specimens from 393 patients with confirmed NSCLC were subjected to immunostaining. Among the NSCLC specimens, 163 (38.1%) were PD-L1 positive. Of these, 82 specimens (50.3%) had low PD-L1 expression levels (1–49%) and 81 (49.7%) had high PD-L1 expression levels (>50%).

### 3.2. Correlation between Patient Characteristics and PD-L1 Expression

Based on immunostaining for PD-L1, patients were divided into two groups: negative (<1%) and positive (≥1%). The association between the clinical characteristics of the 428 patients and PD-L1 expression was evaluated via univariate analysis (Table 1), which revealed that PD-L1-positive tumors were significantly more frequent among male patients (47.0%) than among female patients (25.1%; *p* < 0.0001), among smokers (50.5%) than among never smokers (27.8%; *p* < 0.0001), and among patients harboring wild-type *EGFR* (51.9%) than among those harboring mutant *EGFR* (24.5%; *p* < 0.0001). Moreover, PD-L1 positivity was significantly more frequent in the presence (N1/N2/N3, 48.4%) than in the absence of lymph node metastasis (N0, 33.8%; *p*=0.005) and in SCC (64.2%) rather than in ADC patients (28.5%; *p* < 0.0001). Furthermore, the PD-L1-positive NSCLC patients had significantly higher SUVmax values than their PD-L1-negative counterparts (*p* < 0.0001). On multivariate analysis, a high SUVmax (*p* < 0.0001 and the SCC (*p*=0.002) condition were significantly associated with PD-L1 positivity (*p* < 0.0001).

Among ADC patients (Table 2), PD-L1 positivity was significantly more frequent in male patients (*p*=0.027), smokers (*p*=0.027), stage II/III/IV patients (*p*=0.008), patients with lymph node metastasis (*p* < 0.0001), carriers of wild-type *EGFR* (*p*=0.004), and at higher SUVmax values (*p* < 0.0001). On multivariate analysis, higher SUVmax and the presence of lymph node metastasis and wild-type EGFR were independent predictors of PD-L1 positivity. In the SCC group (Table 3), SUVmax was significantly higher in PD-L1-positive than in PD-L1-negative patients (*p*=0.006). No significant association between PD-L1 expression and additional clinicopathological characteristics was observed.

### 3.3. Correlation between ^18^F-FDG Uptake and PD-L1 Expression

**Figure 2:**
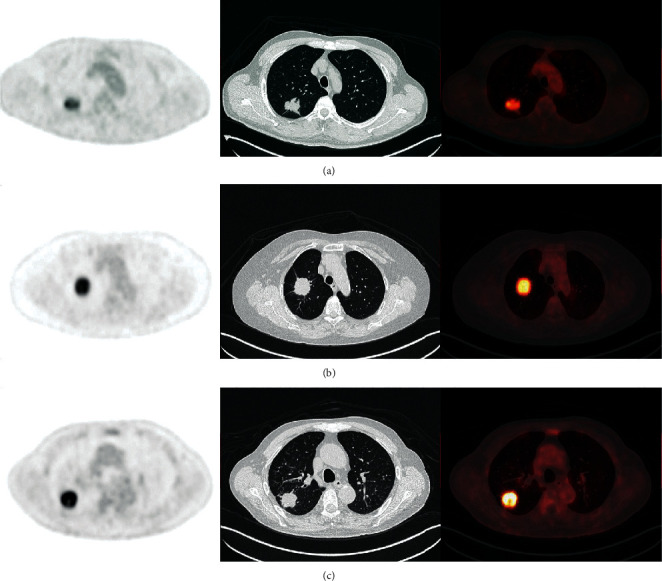
Representative images of ^18^F-fluorodeoxyglucose positron emission tomography/computed tomography (PET/CT), showing variations in the maximum standardized uptake (SUVmax) value in relation to the expression level of programmed cell death ligand-1 (PD-L1). (a) PET/CT images showing FDG uptake by PD-L1-negative adenocarcinoma (ADC) (SUVmax 6.91, size 3.0 cm, and PD-L1 expression < 1%). (b) PET/CT images showing FDG uptake by ADC with low PD-L1 expression (SUVmax 11.37, size 3.1 cm, and PD-L1 expression 1–49%). (c) PET/CT images showing intense FDG uptake by ADC with high PD-L1 expression (SUVmax 19.49, size 3.0 cm, and PD-L1 expression > 50%).

**Figure 3:**
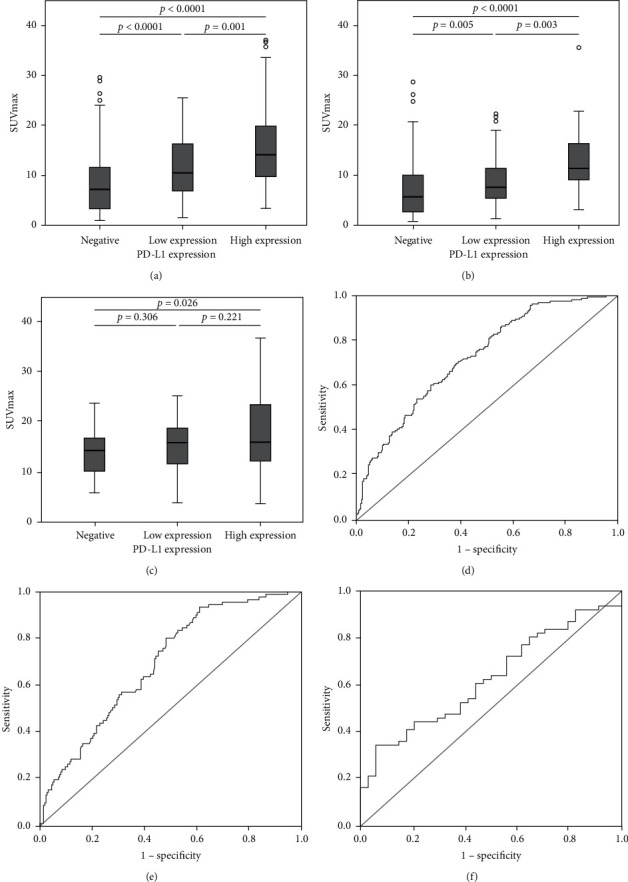
The correlation between maximum standardized uptake value (SUVmax) and programmed cell death ligand-1 (PD-L1) expression. (a) In NSCLC, the SUVmax values of PD-L1-negative (<1%), -low expressing (1%–49%), and -high-expressing (>50%) tumors were 8.22 ± 5.77, 11.63 ± 6.18, and 15.52 ± 7.72, respectively. (b) In ADC, the SUVmax values of PD-L1-negative (<1%), -low expressing (1%–49%), and -high-expressing (>50%) tumors were 7.21 ± 5.15, 9.24 ± 5.25, and 12.91 ± 6.41, respectively. (c) In SCC, the SUVmax values of PD-L1-negative (<1%), -low expressing (1%–49%), and -high-expressing (>50%) tumors were 14.25 ± 4.85, 15.90 ± 5.75, and 18.87 ± 8.59, respectively. Receiver operating characteristic curve analysis of the ability of SUVmax to predict PD-L1 expression levels. The area under the curve for non-small-cell lung cancer (d), adenocarcinoma (e), and squamous cell carcinoma (f) was 0.726 (95% confidence interval 0.679–0.774; *p* < 0.0001), 0.694 (95% confidence interval 0.634–0.755; *p* < 0.0001), and 0.625 (95% confidence interval 0.513–0.738; *p*=0.044), respectively.

We compared the SUVmax between the PD-L1-positive and the PD-L1-negative group observed significant differences among NSCLC (13.55 ± 7.23 vs. 8.22 ± 5.77; *p* < 0.0001), ADC (10.89 ± 6.05 vs. 7.21 ± 5.15; *p* < 0.0001), and SCC (17.41 ± 7.42 vs. 14.25 ± 4.85; *p*=0.006) patients. The results are shown in Tables 1–3. Furthermore, significant differences were observed between groups with high PD-L1 expression and low PD-L1 expression levels for NSCLC (15.52 ± 7.72 vs. 11.63 ± 6.18; *p*=0.001) and ADC (12.91 ± 6.41 vs. 9.24 ± 5.25; *p*=0.003), respectively. In the SCC group, no significant association between groups with high PD-L1 expression and low PD-L1 expression levels was observed. The quantitative differences are shown in Figures 2 and 3(a)–3(c).

In the NSCLC group, the area under curve (AUC) was 0.726 (95% CI, 0.679–0.774; *p* < 0.0001) (Figure 3(d)), and the sensitivity, specificity, positive-predictive value, negative-predictive value, and accuracy in predicting PD-L1 positivity were 86.7%, 44.7%, 49.7%, 84.3%, and 60.9%, respectively. In the ADC group, the AUC was 0.694 (95% CI, 0.634–0.755; *p* < 0.0001) ((Figure 3(e)), and the abovementioned indicators of diagnostic performance were 93.4%, 39.0%, 37.9%, 93.7%, and 54.5%, respectively. In the SCC group, the AUC was 0.625 (95% CI, 0.513–0.738; *p*=0.044) ((Figure 3(f)), and the aforementioned indicators were 34.4%, 94.1%, 91.3%, 44.4%, and 55.8%, respectively.

## 4. Discussion

Immune checkpoint-targeted therapies have changed the therapeutic landscape of NSCLC. Among patients with advanced NSCLC and PD-L1 positivity, antibodies that block the PD-L1 protein improve survival [7, 8]. In clinical practice, however, high-quality tissues from these patients with advanced diseases are not always available for analyzing PD-L1 expression. Therefore, in clinical practice, the presence of a complementary noninvasive biomarker that can predict the PD-L1 expression status would be valuable for patients with advanced NSCLC. In the present cohort, the SUVmax was significantly higher in PD-L1-positive than in PD-L1-negative NSCLC patients. Furthermore, the SUVmax was significantly higher among patients with high PD-L1 expression levels than among those with low PD-L1 expression levels in the NSCLC (*p*=0.001) and the ADC (*p*=0.003) groups. In addition, we examined the association between PD-L1 expression and various clinicopathological features. PD-L1 was expressed in 28.5% of lung adenocarcinomas and 64.2% of lung squamous cell cancers. In ADC patients, PD-L1 positivity was associated with a wild-type EGFR status (*p*=0.046) and with the presence of lymph node metastasis (*p*=0.022). These results are consistent with those of previous studies on NSCLC patients [9–15, 23, 24].

In malignant tumors, the association between PD-L1 expression and ^18^F-FDG uptake was not consistent. Chen et al. [20] reported that MTV and TLG are negative predictors of PD-L1 expression in squamous cell carcinoma of the head and neck, whereas the SUVmax did not differ upon stratification based on the PD-L1 expression status. de Heer et al. [21] reported that the PD-L1 expression status in melanoma was not correlated with any of the PET parameters. Moreover, two studies reported that the PET parameters were significantly associated with PD-L1 expression in both oral squamous cell carcinoma and bladder cancer [19, 22]; however, their cohorts were relatively small, with less than 100 patients. Therefore, their results warrant careful confirmation through studies with larger patient cohorts. Furthermore, the results were inconsistent with those of NSCLC. Two different studies failed to identify an association between the SUVmax and PD-L1 expression [16, 17]. However, their patient cohorts were small, with 55 and 49 lung cancer patients [16, 17], respectively. Other studies have reported that the SUVmax was higher in PD-L1-positive NSCLC patients [9–15]. Only one of these studies has a larger cohort size than the present cohort [11]. Finally, a meta-analysis identified only a weak correlation between SUVmax and PD-L1 expression in lung cancer, concluding that the SUVmax cannot be considered a surrogate marker for the PD-L1 status [18]. To our knowledge, most of these studies only included patients undergoing surgical resection [9–16]. In these studies, the cohort of advanced NSCLC patients was very small. Only Jreige et al. [17] obtained tumor tissues either via biopsy or through surgical resection from 49 patients with NSCLC, representing a small sample size. The present cohort included a larger number of patients with advanced NSCLC (123 patients with III/IV stage tumors). The present results reveal a significant correlation between ^18^F-FDG uptake and PD-L1 expression levels. We believe that our data are reliable, as they were obtained with a relatively large sample size (428 patients) and from a higher number of advanced NSCLC patients, relative to previous studies.

Several studies have reported that in NSCLC, PD-L1 positivity is correlated with high ^18^F-FDG uptake [9–15]. However, in those studies, PD-L1 expression in tumor cells was assessed through different PD-L1 IHC assays, and different cutoff values were defined for PD-L1 positivity. Zhang et al. [9] reported that in SCC, PD-L1 expression levels (cutoff, 5%), assessed through immunostaining with the 28-8 antibody, are significantly correlated with the SUVmax. Moreover, ^18^F-FDG uptake was significantly correlated with PD-L1 expression (cutoff, 10%) (as assessed by using 28-8 and E1L3N antibodies) in ADC patients [13]. Furthermore, Kasahara et al. [14] reported that in SCC, a high SUVmax on ^18^F-FDG PET is associated with PD-L1 positivity (cutoff, 11%), as revealed through the E1L3N assay. Three other studies reported that the SUVmax was an independent predictor for PD-L1 positivity (cutoff, 5%), as revealed through the SP142 assay [10–12]. Furthermore, Wu et al. [15] reported that among patients with pulmonary sarcomatoid carcinoma, the SUVmax was markedly higher when PD-L1 positivity was ≥50% rather than <50%. No studies have investigated the differences in ^18^F-FDG uptake between patients with high PD-L1 expression (≥50%) and low PD-L1 expression (1–49%) levels. The present results show that the SUVmax of the PD-L1 high-expression group was significantly higher than that of the low-expression group in both the NSCLC (*p*=0.001) and the ADC (*p*=0.003) group. It may hence be possible to provide more information regarding the first-line immunotherapy decisions for patients with advanced NSCLC.

Thus far, four PD-L1 assays have been approved by the US Food and Drug Administration (FDA) and the European Medicines Agency (EMA) for clinical use among urothelial carcinoma patients, i.e., based on the Dako 28-8 and 22C3 and the Vantana SP142 and SP263 monoclonal antibodies. We performed immunostaining to evaluate PD-L1 expression in 428 NSCLC patients, using the SP142 monoclonal antibody. However, in previous studies, PD-L1 expression in lung cancer cells was assessed through different PD-L1 immunostaining assays. Three studies have used the SP142 monoclonal antibody [10–12]. Herein, the frequency of PD-L1-positive tumors was higher than those reported previously using the SP142 antibody, most probably owing to the fact that while in the latter studies, a 5% threshold was applied to define PD-L1 positivity, and we used a cutoff value of 1%. Rittmeyer et al. [25] reported that atezolizumab provides survival benefits in previously treated NSCLC patients in accordance with the 1% cutoff value, and this improvement was associated with PD-L1 expression in tumor cells, as assessed via the SP142 PD-L1 immunostaining assay. Takada et al. [26] reported that surgically resected, PD-L1-positive ADC and NSCLC patients had a worse prognosis than their PD-L1-negative counterparts at the 1% cutoff value. Moreover, the 1% cutoff value for assessing PD-L1 expression via the SP142 PD-L1 immunostaining assay was a sensitive indicator to postoperatively predict the patient prognosis [27]. Therefore, we considered that a 1% cutoff value would be acceptable for dichotomizing PD-L1 expression in the present study. We demonstrated that when the SP142 PD-L1 immunostaining assay was used, a high SUVmax was significantly associated with PD-L1 positivity in patients at the 1% cutoff value, as well as at the 5% cutoff value.

This study shows that different clinicopathological parameters were differently associated with PD-L1 expression; as determined via univariate and multivariate analyses, the SUVmax had the highest odds ratio (OR) among all other parameters (5.15 in NSCLC, 9.07 in ADC, and 8.4 in SCC). A high negative-predictive value was obtained in NSCLC and ADC, and a high positive-predictive value was obtained in SCC. However, herein, considering only the SUVmax, the accuracy in predicting PD-L1 expression levels was not very high, and the AUC was 0.726, 0.694, and 0.625 for NSCLC, ADC, and SCC, respectively. In further studies, we intend to combine the SUVmax and other parameters with significant ORs in a combined predictive model to increase the accuracy, e.g., the presence of lymph node metastases and the morphological findings on CT. Furthermore, the cutoff values of SUVmax obtained through ROC curve analysis were 6.06, 4.07, and 20.72 for NSCLC, ADC, and SCC patients, respectively, which varied with the pathological subtype. Moreover, previous studies have suggested that in lung cancer, FDG uptake is influenced by the tumor histologic subtype [28, 29]. We reasoned that the application of different cutoffs for different pathological subtypes may be appropriate. These values were higher than those reported previously in the same three patient subgroups [9–14], probably owing to the use of different methods for PD-L1 immunostaining. The underlying reason was speculated to be the large cohort of advanced NSCLC patients in this study. Our data showed that SUVmax was strongly correlated with PD-L1 expression. However, since the cutoff for SUVmax was not optimized, further studies with a more rigorous and detailed design are warranted.

This study has several limitations. First, this was a retrospective, single-center study. Second, only the SP142 PD-L1 immunostaining assay was used to assess PD-L1 expression in tumor cells. Büttner et al. [30] reported that the highest concordance and interobserver reproducibility were achieved with 28-8, 22C3, and SP263 clinical assays, whereas a lower PD-L1 expression levels were detected via the SP142 assay. Further studies are required to establish the comparability among various PD-L1 immunostaining analyses. Third, NSCLC is a heterogeneous condition comprising various histopathologic patterns. Different pathological NSCLC subtypes exhibit distinct FDG metabolic activities, which may partly account for the contradictory results reported by previous studies. Therefore, subgroup analyses are necessary to establish specific cutoff values in accordance with different pathological subtypes, stage, and to the use of different assays.

## 5. Conclusions

In conclusion, this study shows that in NSCLC, the SUVmax for ^18^F-FDG PET/CT is significantly associated with that of PD-L1 expression, at the 1% cutoff value for assessing through immunostaining with the SP142 antibody. Unlike previous studies, which mostly investigated patients with early-stage disease, this study is the first to assess patients with early and substantially advanced NSCLC. Moreover, this study shows that the SUVmax was higher in the group with high PD-L1 expression than in the low PD-L1 expression group of NSCLC and ADC patients. We speculated that the SUVmax is increased upon PD-L1 upregulation. Thus, determination of the SUVmax may help in identifying patients potentially benefiting from treatment with PD-L1 inhibitors.

## Figures and Tables

**Table 1:** Univariate and multivariate analysis of the relationship between programmed death ligand-1 expression and clinicopathological characteristics of non-small-cell lung cancer.

Clinical factors	Total		PD-L1 expression				Univariate analysis		Multivariate analysis	
	No.	%	Negative		Positive		OR (95% CI)	*p* value	OR (95% CI)	*p* value
	428	100.0	265	61.9%	163	38.1%				
Age (*y*), median (IQR)	62 (56–68)						1.17 (0.79–1.73)	0.438		
<62	194	45.3	124	63.9%	70	36.1%	Reference			
≥62	234	54.7	141	60.3%	93	39.7%				
Sex							2.64 (1.74–4.03)	0.000	1.19 (0.61–2.29)	0.613
Male	253	59.1	134	53.0%	119	47.0%				
Female	175	40.9	131	74.9%	44	25.1%	Reference			
Smoking history							2.65 (1.78–3.97)	0.000	1.22 (0.65–2.29)	0.543
Never	234	54.7	169	72.2%	65	27.8%				
Former/current	194	45.3	96	49.5%	98	50.5%	Reference			
Size (cm), median (IQR)	3.0 (2.4–4.1)						1.41 (0.94–2.08)	0.094		
<3.0	198	46.3	131	66.2%	67	33.8%	Reference			
≥3.0	230	53.7	134	58.3%	96	41.7%				
Pathological stage							1.21 (1.00–1.47)	0.056		
I	230	53.7	158	68.7%	72	31.3%				
II	75	17.5	35	46.7%	40	53.3%				
III	93	21.7	53	57.0%	40	43.0%				
IV	30	7.0	19	63.3%	11	36.7%				
T stage							1.20 (0.98–1.47)	0.086		
T1	214	50.0	144	67.3%	70	32.7%				
T2	134	31.3	77	57.5%	57	42.5%				
T3	44	10.3	21	47.7%	23	52.3%				
T4	36	8.4	23	63.9%	13	36.1%				
Lymph node metastases							1.84 (1.21–2.81)	0.005	1.40 (0.87–2.26)	0.171
N0	302	70.6	200	66.2%	102	33.8%	Reference			
N1/N2/N3	126	29.4	65	51.6%	61	48.4%				
Metastatic							0.94 (0.43–2.02)	0.868		
M0	398	93.0	246	61.8%	152	38.2%	Reference			
M1	30	7.0	19	63.3%	11	36.7%				
SUVmax, median (IQR)	9.42 (4.62–14.49)						5.15 (3.09–8.57)	0.000	2.90 (1.64–5.12)	0.000
<6.06	140	32.7	118	84.3%	22	15.7%	Reference			
≥6.06	288	67.3	147	51.0%	141	49.0%				
Histological subtype							3.99 (2.62–6.09)	0.000	2.12 (1.30–3.45)	0.003
ADC	319	74.5	228	71.5%	91	28.5%				
SCC	95	22.2	34	35.8%	61	64.2%				
Other	14	3.3	3	21.4%	11	78.6%				
EGFR mutation status							3.32 (2.20–5.00)	0.000	1.60 (0.96–2.68)	0.074
Mutation	216	50.5	163	75.5%	53	24.5%	Reference			
Wild type	212	49.5	102	48.1%	110	51.9%				

PD-L1, programmed cell death ligand-1; SUVmax, maximum standardized uptake value; SCC, squamous cell carcinoma; EGFR, epidermal growth factor receptor; IQR, interquartile range.

**Table 2:** Univariate and multivariate analysis of the relationship between programmed death ligand-1 expression and clinicopathological characteristics of pulmonary adenocarcinoma.

Clinical factors	Total		PD-L1 expression				Univariate analysis		Multivariate analysis	
	No.	%	Negative		Positive		OR (95% CI)	*p* value	OR (95% CI)	*p* value
	319	100.0	228	71.5%	91	28.5%				
Age (*y*)							1.13 (0.69–1.83)	0.632		
<61	154	48.3	112	72.7%	42	27.3%				
≥61	165	51.7	116	70.3%	49	29.7%				
Sex							1.73 (1.06–2.84)	0.027	1.02 (0.48–2.19)	0.952
Male	151	47.3	99	65.6%	52	34.4%				
Female	168	52.7	129	76.8%	39	23.2%	Reference			
Smoking history							2.01 (1.21–3.33)	0.007	1.55 (0.71–3.37)	0.272
Never	215	67.4	164	76.3%	51	23.7%	Reference			
Former/current	104	32.6	64	61.5%	40	38.5%				
Size (cm)							1.24 (0.76–2.03)	0.396		
<3.2	194	60.8	142	73.2%	52	26.8%				
≥3.2	125	39.2	86	68.8%	39	31.2%				
Pathological stage							1.35 (1.08–1.69)	0.008	0.70 (0.45–1.08)	0.110
I	186	58.3	145	78.0%	41	22.0%				
II	37	11.6	23	62.2%	14	37.8%				
III	68	21.3	42	61.8%	26	38.2%				
IV	28	8.8	18	64.3%	10	35.7%				
T stage							1.13 (0.87–1.48)	0.345		
T1	176	55.2	133	75.6%	43	24.4%				
T2	98	30.7	64	65.3%	34	34.7%				
T3	22	6.9	13	59.1%	9	40.9%				
T4	23	7.2	18	78.3%	5	21.7%				
Lymph node metastases							2.83 (1.69–4.73)	<0.0001	3.01 (1.17–7.74)	0.022
N0	224	70.2	175	78.1%	49	21.9%	Reference			
N1/N2/N3	95	29.8	53	55.8%	42	44.2%				
Metastatic							1.44 (0.64–3.25)	0.380		
M0	291	91.2	210	72.2%	81	27.8%	Reference			
M1	28	8.8	18	64.3%	10	35.7%				
SUVmax							9.07 (3.80–21.64)	<0.0001	7.52 (3.03–18.65)	<0.0001
<4.07	95	29.8	89	93.7%	6	6.3%				
≥4.07	224	70.2	139	62.1%	85	37.9%				
EGFR mutation status							2.10 (1.27–3.48)	0.004	1.77 (1.01–3.11)	0.046
Mutation	211	66.1	162	76.8%	49	23.2%	Reference			
Wild type	108	33.9	66	61.1%	42	38.9%				

PD-L1, programmed cell death ligand = 1 SUVmax, maximum standardized uptake value; EGFR, epidermal growth factor receptor.

**Table 3:** The relationship between programmed death ligand-1 expression and clinicopathological characteristics of pulmonary squamous cell carcinoma.

Clinical factors	Total		PD-L1 expression				Univariate analysis	
	No.	%	Negative		Positive		OR (95% CI)	*p* value
	95	100.0	34	35.8%	61	64.2%		
Age (*y*)							0.564 (0.24–1.34)	0.193
<64	42	44.2	12	28.6%	30	71.4%	Reference	
≥64	53	55.8	22	41.5%	31	58.5%		
Sex							0.89 (0.08–10.24)	0.928
Male	92	96.8	33	35.9%	59	64.1%	Reference	
Female	3	3.2	1	33.3%	2	66.7%		
Smoking history							2.27 (0.59–8.80)	0.234
Never	14	14.7	3	21.4%	11	78.6%		
Former/current	81	85.3	31	38.3%	50	61.7%	Reference	
Size (cm)							0.66 (0.28–1.53)	0.335
<4.2	51	53.7	16	31.4%	35	68.6%	Reference	
≥4.2	44	46.3	18	40.9%	26	59.1%		
Pathological stage							0.72 (0.42–1.21)	0.211
I	39	41.1	12	30.8%	27	69.2%		
II	34	35.8	11	32.4%	23	67.6%		
III	21	22.1	11	52.4%	10	47.6%		
IV	1	1.1	0	0.0%	1	100.0%		
T stage							0.92 (0.61–1.39)	0.696
T1	35	36.8	11	31.4%	24	68.6%		
T2	30	31.6	12	40.0%	18	60.0%		
T3	19	20.0	7	36.8%	12	63.2%		
T4	11	11.6	4	36.4%	7	63.6%		
Lymph node metastases							0.68 (0.27–1.72)	0.417
N0	69	72.6	23	33.3%	46	66.7%	Reference	
N1/N2/N3	26	27.4	11	42.3%	15	57.7%		
Metastatic								1.000
M0	94	98.9	34	36.2%	60	63.8%	Reference	
M1	1	1.1	0	0.0%	1	100.0%		
SUVmax							8.4 (1.83–38.53)	.006
<20.72	72	75.8	32	44.4%	40	55.6%		
≥20.72	23	24.2	2	8.7%	21	91.3%		
EGFR mutation status								0.999
Mutation	2	2.1	0	0.0%	2	100.0%	Reference	
Wild type	93	97.9	34	36.6%	59	63.4%		

PD-L1, programmed cell death ligand-1; SUVmax, maximum standardized uptake value; EGFR, epidermal growth factor receptor.

## Data Availability

The datasets used and/or analyzed during the current study are available from the corresponding author on reasonable request.

## Abbreviations

^18^F-FDG PET/CT: ^18^F-fluorodeoxyglucose positron emission tomography/computed tomography
PD-L1: Programmed death ligand 1
EGFR: Epidermal growth factor receptor
NSCLC: Non-small cell lung cancer
SUVmax: Maximal standard uptake value
ADC: Pulmonary adenocarcinoma
SCC: Pulmonary squamous cell carcinoma.
